# The Detection and Phylogenetic Analysis of Bovine Hepacivirus in China

**DOI:** 10.1155/2018/6216853

**Published:** 2018-05-31

**Authors:** Yu Deng, Su Hua Guan, Silu Wang, Guiying Hao, Thomas Bruun Rasmussen

**Affiliations:** ^1^School of Animal Science, Xichang College, Xichang 615013, China; ^2^DTU National Veterinary Institute, Technical University of Denmark, Lindholm, Kalvehave 4771, Denmark

## Abstract

*Hepacivirus* has been identified in cattle in Africa, Europe, and South America. In this survey of bovine hepacivirus (BovHepV) in 131 serum samples from Chinese cattle herds using RT-PCR, five of 131 sera were BovHepV positive, with the infection rate of 3.82%. Phylogenetic analysis based on the partial NS3 coding sequence showed that the BovHepV of the five positive samples clustered with other BovHepV but formed a separate branch. The results indicated that these new BovHepV represent emerging and novel strains. Further investigations are necessary to determine the epidemiology and viral pathogenesis of these BovHepV strains, as well as the potential threat to ruminant and livestock workers in China.

## 1. Introduction

The genus* Hepacivirus* is one of the four genera within the family Flaviviridae. This genus comprises the major human pathogen hepatitis C virus (HCV) and a range of other genetically diverse pathogenic viruses that cause human and animal diseases, such as yellow fever virus, dengue virus, bovine viral diarrhea virus, and classical swine fever virus [1].

HCV causes hepatocellular carcinoma, cirrhosis, and liver failure in humans and chronic HCV infection is a global health problem. It has been estimated that it affects over 184 million people worldwide [2]. HCV-like viruses have been identified in respiratory samples from a domestic dog in the United States in 2011 [3], which was temporarily named as canine hepacivirus, as well as in horses [4–6], rodents [7], bats [8], cows [9], and nonhuman primates [10]. Diverse hepacivirus sequences have been found in nonprimate animals from Africa, Europe, Central American, South America, and Asia [7–9, 11, 12]. In China, hepacivirus and pegivirus isolates were first detected in domestic horses in 2016 [13]. Recently, the first report of the detection of bovine hepacivirus (BovHepV) in Chinese dairy cattle of a farm in Guangdong Province, Southern China, was published in April 2018 [14].

BovHepV can cause chronic infection without any signs of clinical disease [11]. The previous studies highlighted the importance to search for possible virus reservoirs. Therefore, in this study, we aimed to detect and characterize the genetic diversity of this emerging virus in healthy cattle herds in Sichuan Province, Southwestern China.

## 2. Materials and Methods

### 2.1. Serum Samples

In total, 131 bovine serum samples were collected from three different regions of China (Xichang, Xide, and HuiLi counties) in 2017 and stored immediately at –20°C until their analysis. All samples were obtained from clinically healthy dairy cows.

### 2.2. Viral RNA Extraction and Reverse Transcription- (RT-) PCR

One milliliter of serum sample from each animal was filtered (0.45-*μ*m pore) to remove eukaryotic and bacterial-sized particles and then treated with nucleases to digest non-particle-protected nucleic acid. After DNase treatment, viral RNA was extracted using a TIANamp Virus DNA/RNA Kit (Tiangen, Beijing, China). cDNA was synthesized using a PrimeScript II First Strand cDNA Synthesis kit (TakaRa, Dalian, China). A 310-bp fragment of the NS3 coding region of BovHepV was amplified as described previously [11]. PCR was performed according to the manufacturer's instructions with a 2x Taq PCR MasterMix kit (Tiangen, Beijing, China). The PCR products were purified using a regular gel DNA purification kit (Tiangen, Beijing, China) and then cloned with a Mighty TA-cloning kit (TaKaRa, Dalian, China). Three positive clones were selected from each positive sample and sequenced by Shanghai JieLi Co. Ltd. (Shanghai, China) before their phylogenetic analysis.

### 2.3. Sequence and Phylogenetic Analysis

To determine the evolutionary status of BovHepV circulating in healthy cattle in China, the sequences obtained in this study were compared with hepacivirus reference sequences in the GenBank database, including those from other animal species and human. The nucleotide (nt) and amino acid (aa) alignments were obtained using ClustalW and DNASTAR (version 15.0), respectively. A phylogenetic tree was created based on the nucleotide sequence of the partial NS3 gene using the neighbor-joining method with 1000 bootstrap replicates, Tamura three-parameter algorithm, transitions + transversions, and pairwise deletion in MEGA 7.0 software (bootstrap values below 50% are not shown).

## 3. Results and Discussion

To investigate whether BovHepV is present in healthy cattle in China, 131 bovine serum samples were collected from herds distributed in three different regions of China during 2017. Information about the cattle breeds was not collected. The prevalence of BovHepV was screened by RT-PCR based on the partial NS3 coding region. The results showed that five samples originating from different herds in three different regions were BovHepV positive. Thus, the BovHepV positive rate in this study was determined as 3.82% (5/131) in these healthy cattle from China.

**Figure 1:**
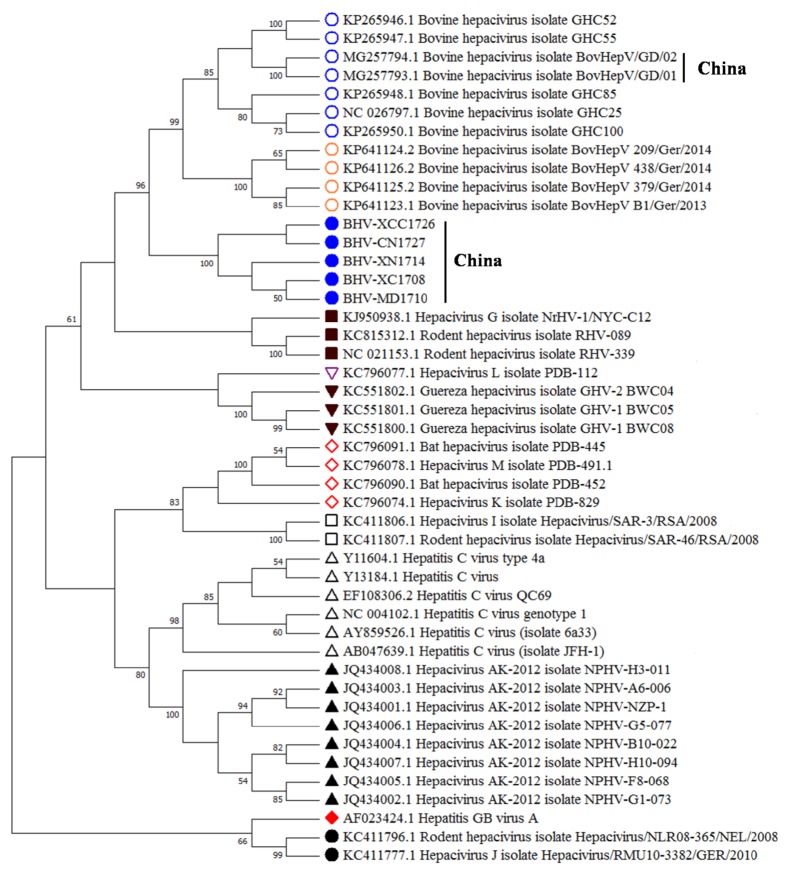
Phylogenetic analysis of the BovHepV isolates identified in this study and other hepaciviruses from human and various animal species. The neighbor-joining tree was constructed based on 310 nt in the coding sequences of NS3 of hepaciviruses. Bootstrap analysis was performed with 1,000 replicates (the numbers close to the branches are percentages), the Tamura three-parameter model, and transitions + transversions. Sequences from the GenBank database are cited with their accession numbers. Sequences are identified as follows: empty blue circle, bovine hepacivirus; empty orange circle, novel bovine hepacivirus; filled blue circle, BovHepV in this study; filled black inverted triangle, hepacivirus from black-and-white colobus; filled black square, hepacivirus from* Rattus norvegicus*; empty black inverted triangle, hepacivirus from* Hipposideros vittatus*; empty black square, hepacivirus from* Rattus norvegicus*; empty red diamond, hepacivirus from bat; empty black triangle, hepacivirus from human; filled black triangle, hepacivirus from horse; filled black circle, hepacivirus from rodent; filled red diamond, hepatitis GB virus A.

The sequences have been submitted in GenBank under accession numbers MG893562 to MG893566. The five variants were designated as BHV-XC1708, BHV-MD1710, BHV-XN1714, BHV-XCC1726, and BHV-CN1727 (Figure 1).

In 2015, the prevalence of hepacivirus BovHepV was first determined by investigating 106 serum samples collected from African cattle [9]. In the same year, a novel BovHepV was detected in German bovine serum samples, where 1.6% (5/320) of the individual animals and 3.2% (5/158) of the investigated cattle herds were infected [11]. A novel BovHepV was also identified in Brazilian cattle [12]. The first detection of two BovHepV strains (BovHepV/GD01 and BovHepV/GD02) in China were reported in 8 out of 102 serum samples from dairy cows of a farm in southeast of China [14]. In the present study, we assessed the prevalence and molecular characteristics of BovHepV in healthy cattle in southwest of China.

We evaluated the relatedness between the BovHepV obtained in this study and the previously reported BovHepV sequences deposited in the GenBank database. The nt and aa sequences in the partial NS3 coding region (310 nt) obtained from the five serum samples in this study were aligned with those of previously reported hepaciviruses, including those from humans and other animals. The sequence homology between our sequences was remarkably high, i.e., ranging 97.7%–99.0% for nt and 99.0% to 100% for aa sequence (data not shown). However, these sequences shared low similarity with the BovHepV reference strains (GHC25, GHC52, GHC55, and GHC100), i.e., ranging from 73.6% to 75.2% for nt sequences and 84.5%–88.3% for aa sequences and with BovHepV_209/Ger/2014, BovHepV_379/Ger/2014, BovHepV_438/Ger/2014, BovHepV_438/Ger/2014, and BovHepV_B1/Ger/2013 [11], ranging from 72.0% to 74.6% and 85.4–86.4% for aa sequences (data not shown).

In addition, we analyzed the identity of nucleotide sequences of the NS3 gene between the previously reported China strains (BovHepV/GD01 and BovHepV/GD02) and our sequences. The results demonstrated that the similarity between these sequences ranged from 72.0% to 73.3% (data not shown).

The NS3 gene is located in a highly conserved region and it is often used in phylogenetic analyses of hepacipiviruses [15]. Therefore, in this study, the phylogenetic tree was constructed based on the partial NS3 coding sequences. The result showed that five Chinese BovHepV sequences clustered with reference BovHepV and temporarily named novel BovHepV reference samples, but distantly to Guereza hepacivirus isolates (GHV-2 BWC04, BWC05, and BWC08) and other hepaciviruses (Figure 1). Interestingly, our five Chinese BovHepV sequences were grouped into a separate clade in contrast to two Chinese BovHepV strains (BovHepV/GD01 and BovHepV/GD02), as shown in Figure 1. Noticeably, our samples are from different farms of different regions in Southwestern China, while BovHepV/GD01 and BovHepV/GD02 were detected in Southeastern China. Together, the phylogenetic reconstruction clearly demonstrated that BovHepV exhibits genetic diversity where the genetic groups have different geographical patterns in China. Combined with the homology analysis, our results suggest that the emerging BovHepV detected in this study differed from the BovHepV sequences deposited previously in the GenBank database.

It has been reported that the NS3 has a critical role in host-virus coevolution [16, 17]. The phylogenetic tree constructed based on NS3 showed that independent clusters were assembled for the same hepacivirus strains from the same species. Thus, changes in the nucleotides within NS3 probably yielded lineages that could infect the same type of viral host but not other lineages, as suggested in a previous study [18].

In conclusion, phylogenetic analysis based on the partial NS3 protein showed that our BovHepV sequences were genetically distant from previously reported BovHepV isolates, and thus our sequences could be new emerging BovHepV strains. However, due to the limited sample size, our serum samples might not have been fully representative of the cattle in China. Therefore, further studies should be conducted to determine the prevalence, genetic characteristics, and pathogenesis of BovHepV in China.

## Data Availability

The data used to support the findings of this study are available from the corresponding author upon request.
